# Exogenous folates stimulate growth and budding of *Candida glabrata*

**DOI:** 10.15698/mic2015.05.202

**Published:** 2015-05-01

**Authors:** Afsaneh Porzoor, Ian G. Macreadie

**Affiliations:** 1 School of Applied Sciences, RMIT University, Bundoora, Victoria 3083, Australia.

**Keywords:** Candida glabrata, folate, proliferation, quiescence, unscheduled cell division

## Abstract

Folate, vitamin B9, is well recognized as being essential for cell growth. The utilization of folate is common to all cells, but the source of it may be quite different. For example, mammalian cells depend on exogenous uptake of folates, while plants and microbes can synthesize them. There has been little consideration of uptake of folate in microbial cells, and studies on the effects of folates in mammalian cells, where conditions are restricted. This study shows that exogenous folates (folic acid or folinic acid), causes *Candida glabrata* cells suspended in water alone to undergo two cycles of cell division and to form multiple buds. The effect was limited to cells in the stationary phase and more profound in quiescent cells. These data indicate a novel response of yeast to folates that may increase the utility of yeast as a model to study folate transport and signaling.

## INTRODUCTION

Folates are essential for all life. Also known as a family of pteroylglutamates (which includes folate - folic acid - vitamin B9), folates are necessary for one carbon metabolism, and for facilitating the production of proteins and DNA. Mammalian cells cannot synthesize folates and are dependent on folates in their diet, utilising receptors and transporters to deliver them into cells. Plants and microbes synthesise folates *de novo* and therefore should have no need for the uptake of folates and for folate transporters and receptors. Yeast with folate synthesis genes deleted, demonstrate that folate, or the cellular processes depending on it are essential 1,2. These yeast deletion strains can be grown in media containing folinic acid or folic acid, or through the addition of adenine, methionine, histidine and thymidine monophosphate, using a *TUP1* strain that contains a thymidine uptake mutation.

Information on uptake of folates comes from a variety of mammalian cell studies, including naturally occurring human mutations that inform us about the importance of uptake of folates for proper embryonic development, and prevention and treatment of disease throughout life, including age-related diseases like Alzheimer’s disease. Microbial models have been important in providing fundamental information about human health and disease, yet insights into uptake of folates have not occurred in microbial models due to an assumption that they would be irrelevant, since they do not rely on exogenous folates. In this study we have focused on the effects of exogenous folates on yeast, since yeast can be extensively manipulated in terms of its environment and genes. For example, yeast can be maintained for long periods in a quiescent state, simply by holding them in aqueous suspension. Our work in this study focused on the effects of added folates on yeast suspended in water.

## RESULTS

### Folates induce cells in stationary phase to proliferate and bud

**Figure 1:**
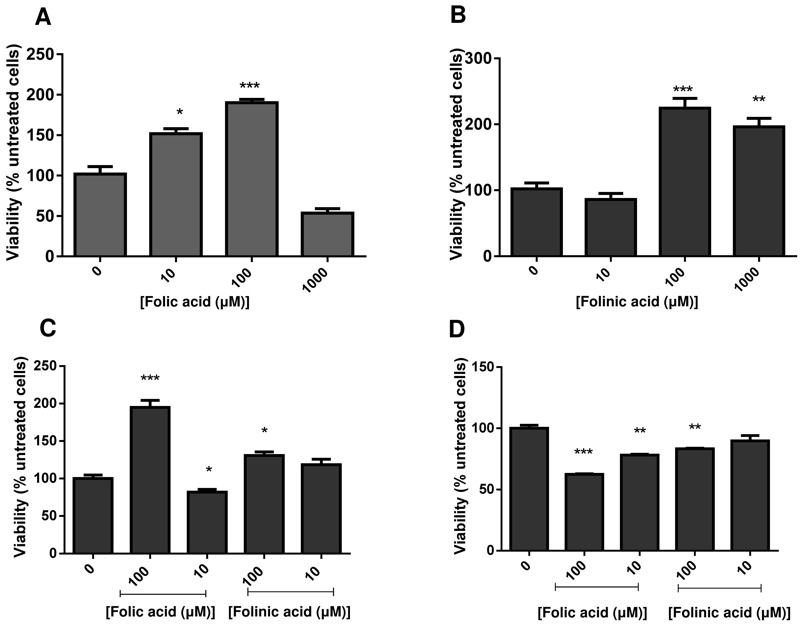
FIGURE 1: Effect of folic acid **(A) ** and folinic acid **(B) ** on the survival of stationary phase yeast. Cells (10^3^ cells/ml) were incubated overnight at 30°C with shaking at 200 rpm. Viability was estimated by determination of CFUs. The viability of quiescent **(C) ** and non-quiescent **(D) ** fractions of stationary phase yeast in the presence of 10 μM and 100 μM of folic acid and folinic acid was compared with the control. (*P < 0.05, **P < 0.01, ***P < 0.005). All data are shown as mean +/- SEM.

*C. glabrata *cells were grown to stationary phase, and then suspended in water with folic or folinic acid in concentrations up to 1 mM. The addition of folic and folinic acid caused a significant increase in the viability within the first 24 h measured by CFUs (colony forming units) (Fig. 1 A,B). Treatments with 10 and 100 µM folic acid (Fig. 1A) or 100 and 1000 µM folinic acid (Fig. 1B) led to significant increases in CFUs. Our studies have focused on the lower concentrations that caused an increase in CFUs. The effect on cell proliferation caused by folates was limited to the cells in stationary phase and no effect was detected on cells growing in exponential phase (data not shown).

The cells were fractionated in quiescent and non-quiescent populations on a Percoll gradient for further examination. Induction of cell proliferation by folates was limited to quiescent cells with a significant increase in the number of cells in the presence of 100 μM of folic acid (P < 0.01) and folinic acid (P < 0.05). A non-quiescent sub-fraction of cells did not behave similarly: folates seemed to reduce their viability (Fig. 1 C,D).

**Figure 2:**
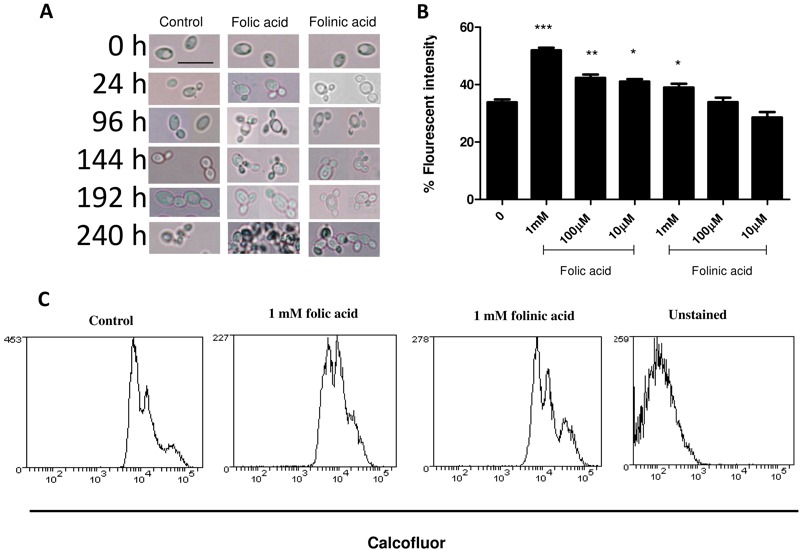
FIGURE 2: Effect of folates on yeast budding. **(A) **Light microscopy images of *C. glabrata *in the presence of 100 µM folic acid and folinic acid compared to the control for up to 10 days. (Scale bar = 10 µm). Calcofluor staining of the bud scars in stationary phase yeast incubated in the presence of 1000, 100 and 10 µM folic acid and folinic acid, analysed by flow cytometry. **(B) **Histograms represent the results from three independent experiments. (*** P < 0.005, ** P < 0.01,* P < 0.05). (C) Flow cytometry profile of cell populations in the presence of 1 mM folic and folinic acid.

Stationary phase cultures were also examined microscopically after folic and folinic acid treatments for a period of 10 d. Prior to treatment, the population consisted of over 90% unbudded cells and approximately 10% budded cells. After 24 h incubation in water, most cells grew at least one bud, while cells treated with folate budded synchronously and had the ability to form multiple buds (Fig. 2A). Some cells grew three or four buds, while no folate addition resulted in no increase in budding. After prolonged incubation in water these yeast cells became larger and more transparent, causing difficulties with cell imaging. Interestingly, both axial and bipolar forms of budding were viewed after the treatment. Although some cells in the control samples had more than one bud, the total percentage was much lower than those of the treated samples. In particular, it appears that the response operates in a dose-dependent manner: as the concentration of folate increases, more multi-budded cells can be counted compared with lower concentrations. This effect persists and could be viewed for the ten day period in which the cells were monitored (Fig. 2A). Cells also seemed more adherent and became larger and more elongated as the incubation continued.

### Flow cytometry analysis of treated cells

Cell growth in the folate treated cells was also examined by calcofluor white staining of bud scars. In the presence of folic and folinic acid there were dose-dependent increases in the fluorescence intensity (Fig. 2B). The fluorescence intensity increased by 41.4% (P < 0.005), 24.8% (P < 0.01) and 19.0% (P < 0.05) in samples treated with 1000, 100 and 10 µM of folic acid, respectively, compared with the control where 15.6% of cells exhibited fluorescence from calcofluor (Fig. 2B). Folinic acid also increased the fluorescence intensity of the cells but to a lesser extent: values of 26.8%, 21.0% and 19.0% were obtained respectively (Fig. 2 B). These results support our observations from both plate counts and microscopy which showed an increase in the number of cells and particularly the number of multi-budded cells.

Flow cytometry of cells after calcofluor staining showed three distinct populations of *C. glabrata* cells (Fig. 2C). The least stained cells are newly budded cells, and the more highly stained cells have one or more bud scars. It can be seen that treatment with folic or folinic acid led to an increased proportion of the populations with one or more bud scars.

## DISCUSSION 

In the present study we investigated the influence of folates on yeast cells in the absence of nutrients. The exogenous addition of folic and folinic acid induced cell proliferation, suggesting an effect of folates on the cell cycle, possibly through a cell surface receptor, that would signal cell division processes to commence. Granot and Snyder 3 showed that glucose could induce *S. cerevisiae *suspended in water to enter the cell cycle, however, this was associated with a loss of viability. The effect of folates in this study led to an increase in cell number, except for the addition of a very high level folic acid (1000 µM) which led to a loss of viability: however, survivors from that treatment grew into larger colonies. The differences in such effects are currently unclear, but we speculate that they may relate to the level of signaling. For example, sustained signaling to divide in the absence of nutrition would presumably lead to cell death. We presume that this happens in water with both glucose, and with 1000 µM folic acid.

The budding pattern induced by folates was also notable. *C. glabrata* normally exhibits only axial budding pattern but folates induced both axial and bipolar forms of budding. It is known that yeast actin cytoskeleton is responsible for polarized growth during budding in the course of the cell cycle 4. Although, in haploid cells the absence of sugar have been reported to cause bud switching from axial to a unipolar-distal budding pattern, no study (to our knowledge) has shown the appearance of three to four buds on cells deprived of nutrients while in stationary phase.

Cells in stationary phase are well equipped for survival and have some reserves for growth. This is clearly demonstrated by our observations that such cells can double without need for additional exogenous nutrients. Also previous studies on *S. cerevisiae* have shown the level of intracellular carbohydrate increases during stationary phase but this increase in intracellular storage does not correlate with long term viability of the cells 5,6. The effect of folate appears limited to stationary phase culture further confirming that regulation of gene expression in yeast is different during the stationary phase of the growth cycle compared to the exponential phase. Further questions relate to why folates have this effect, since it seems counterintuitive that yeast, an organism that normally synthesizes its own folate, should respond to exogenous folic or folinic acid.

Additionally we have shown proliferation in the quiescent cells whereas the non-quiescent cells lost their viability in the presence of folate. This result is in agreement with previous work demonstrating the presence of a mature spindle pole body and low ROS in quiescent cells versus a premature spindle pole body and higher level of ROS in non-quiescent cells, making the latter more susceptible to environmental changes 6,7,8.

## MATERIALS AND METHODS

### Yeast strain, media and storage condition

*C. glabrata *ATCC90030 was utilised in this study. Chemicals, solvents and reagents were purchased from Sigma-Aldrich® (Australia) unless otherwise stated. All cells were grown in YEPD (1% yeast extract, 2% peptone and 2% D-glucose) with aeration at 30°C. For short-term storage (<3 weeks), yeast cells were maintained on YEPD solidified media at 4°C. For long-term storage, yeast cells were stored -80°C in YEPD or suitable selective broth and 15% glycerol.

### Stationary and exponential phase growth of yeast 

In order to prepare yeast for stationary phase analysis, cells were grown in YEPD broth with aeration at 30°C for 7 days with shaking (200 rpm). Cells were then pelleted and washed twice and resuspended in filter sterilized H_2_O at the density of 10^5^ cells/ml for further treatment. For yeast in exponential growth phase, yeast cells were grown overnight in YEPD broth and then 1 ml was inoculated into 80 ml of fresh media and incubated at 30°C with shaking (200 rpm) for 2-3 h to reach an OD_600_ of 1.0.

### Fractionation of yeast cells into quiescent and non-quiescent populations 

Percoll density gradients (GE Healthcare) were prepared according to the manufacturer’s protocol and the method described by Allen and colleagues 9 with some modification. Briefly, to form a gradient, 9 ml of Percoll was mixed with one ml of 1.5 M NaCl. This solution was then added to 15 ml Corex tubes and centrifuged at 13,800 rpm for 15 min at 20°C in Beckman Coulter centrifuge (SW41Ti rotor). ~2 x 10^9^ stationary phase yeast cells, which were grown for 7 days at 30°C (200 rpm), were pelleted and resuspended in 1 ml Tris buffer (50 mM, pH 7.5). Around 2 ml of cell suspension was overlaid onto the preformed gradient, and centrifuged at 400 *g* for 60 min in a Beckman Coulter centrifuge (Avanti J25-01). Fractions of quiescent and non-quiescent were collected and washed three times in 40 ml Tris buffer and then resuspended in ddH_2_O for analysis.

### Folic acid and folinic acid treatment of *C. glabrata *

10 mM stock solutions of folic acid and folinic acid were made in water. Folic acid was dissolved with addition of NaOH and pH was adjusted to 7.0 prior to use. All solutions, including the folate stock solutions, were filter sterilized through 0.22 μm syringe filters (Sarstedt). Stationary phase *C. glabrata* were washed and suspended in sterile water to densities of 10^5^ cells/ml for microscopy and flow cytometry, or 10^3^ cells/ml for CFU analyses. For folic acid and folinic acid treatments, the yeast cells were transferred into 96-well culture plates and 10-fold dilutions series of the folates were made to achieve concentrations of 1 mM and lower. Samples in triplicate were treated with 10-1000 µM of folic acid or folinic acid and incubated for a period of up to 10 days. Aliquots were taken every 24 h for microscopy and CFU determination.

### Cell viability assay

Cell viability was determined by plating 100 µl aliquots of cell suspension from both treated and untreated samples on YEPD plates and counting the number of colonies produced after at least 2 days of incubation at 30°C. Colonies were counted and the data presented as a percentage of untreated samples.

### Calcofluor staining of bud scars

Working solutions of 25 µM calcofluor white M2R were prepared from a 5 mM stock solution. Folic acid and folinic acid treated stationary phase *C. glabrata* cells (2 x 10^5 ^cells/ml) were stained with this working solution and incubated for 10 min in the dark at room temperature. Around 30,000 cells per sample were analysed by flow cytometry (FACS, Canto II- BD™) using 351 nm excitation and collecting fluorescence emission with filters at 450/65. Data were recorded and saved as FCS3 files. All data were analysed by WEASEL (WEHI, Parkville, Australia) software.

### Microscopic analysis of cell morphology

Around 10 µl of each sample was taken and using a wet mount method, loaded on a microscopic slide for imaging using oil immersion on an Olympus CX31 light microscope. Images were captured using an Olympus digital camera (E330- Japan).

### Statistical analyses

Prism 5 for windows version 5.04 (GraphPad software, Inc., La Jolla, CA, USA) was used to perform statistical analyses. All data are presented as mean with a standard error of the mean, and differences were compared using unpaired Student’s *t *test and one way ANOVA with *Tukey post hoc *test. Significant differences are indicated by asterisks: *P < 0.05; **P < 0.01; ***P < 0.005.
